# Supplementary material for: The apple detection method based on multimodal features
Source: PLoS One. 2025 Oct 23;20(10):e0334911. doi: 10.1371/journal.pone.0334911 (PMC12548850; doi:10.1371/journal.pone.0334911)
Supplement: Attachment — Submitted filename: comments[1].docx [file pone.0334911.s001.docx]

**Reviewer Comments:**

The article titled *“The Apple Detection Method Based on Multimodal Features”* presents a very interesting and well-structured study. The authors provide a clear and insightful contribution to the field, particularly in the application of multimodal feature integration for fruit detection, which is both timely and relevant.

The methodology is sound, and the results are presented clearly. The use of multiple data modalities enhances detection accuracy, and the analysis demonstrates the potential of the approach in practical agricultural applications. The paper offers a valuable perspective and advances understanding in this area.

While the overall quality of the work is commendable, I have suggested minor corrections to improve clarity and precision in a few sections of the manuscript. These suggestions are included in the attached file.

**Abstract**

**Line 6**

change “improve the enhances” to “improve the detection” or “enhance the performance” , not both.

**Introduction**

**Line 5**

please add recent year report data.

**Line 7-9**

Break into two concise sentences.

**Line 10 -19**

This paragraph provides useful background on traditional methods but can be made more concise.

**Line 18**

Please explain what kind of uncertainty.

**Line 40**

Clarify what “organ segmentation”. Does it mean fruit vs. stem?

**Line 50**

Good transition, but could add a brief example of a depth camera.

**Line 72**

Please merge with the previous sentence for better flow

**Material and methods**

Line 106

The apples images change to apple images it should be singular when use like this.

You may add the reason for choosing this specific camera.

**Line 108**

Nice description, but could explain why its versatile.

**Line 115**

Please explain how these models differ visually from real apples.

**Line 119**

Not all readers may know RGB8, please explain.

**Line 124**

Good job explaining why 30 frames are used. This strengthens the reasoning.

**Line 127**

You wrote 30 frames were merged but did not explain how. Please mention the software/methods.

**Line 139**

Voxel Down Sampling technology consider simplifying.

**Line 157**

Zeros are applying Explain why it is important.

**Line 161-163**

Add one sentence about why this is important.

**Line 183**

Consider explaining why 80% was chosen as the cutoff. Is this standard?

**Line 189**

fruits occluded heavily reword it for smooth flow.

**Line 282**

The feature map of color difference… this line is too long.

**Line 287**

Suppressed could be simplified to reduced.

**Line 289**

Use simple language.

**Line 325**

Correct grammar.

**Line 329**

You could specify how many channels are in final image.

**Line 390 to 395**

Very detailed settings but you can convert it into a table or appendix.

**Line 420 table number 1**

Mention units clearly

**Line 474 to 476**

You can emphasize it why this combination is powerful.

**Line 494 495**

Also explain How this helps in agriculture.

I recommend acceptance of the paper after these minor revisions are made.
