# Supplementary material for: The apple detection method based on multimodal features
Source: PLoS One. 2025 Oct 23;20(10):e0334911. doi: 10.1371/journal.pone.0334911 (PMC12548850; doi:10.1371/journal.pone.0334911)
Supplement: Attachment — Submitted filename: Response to Reviewers.docx [file pone.0334911.s002.docx]

**Author's Response To Reviewer Comments**

PONE-D-25-19188

The apple detection method based on multimodal features

PLOS ONE

We sincerely thank the reviewers for their insightful comments and constructive feedback. Their suggestions have greatly helped us improve the clarity, depth, and overall quality of the manuscript. We have addressed each comment carefully and made the corresponding revisions in the manuscript, as detailed below.

Additional Editor Comments:

The paper requires careful and extensive revision before 2nd review round.

Response: Thank you for your feedback and guidance. We will spare no effort to carry out thorough and meticulous revisions, covering all relevant aspects to meet the requirements for the next review. We will promptly complete the revision work and submit the revised version in a timely manner.

Reviewer's Responses to Questions

**Comments to the Author**

1. Is the manuscript technically sound, and do the data support the conclusions?

The manuscript must describe a technically sound piece of scientific research with data that supports the conclusions. Experiments must have been conducted rigorously, with appropriate controls, replication, and sample sizes. The conclusions must be drawn appropriately based on the data presented.

**Reviewer #1:** Yes

2. Has the statistical analysis been performed appropriately and rigorously?

**Reviewer #1:** N/A

3. Have the authors made all data underlying the findings in their manuscript fully available?

The [PLOS Data policy](http://track.editorialmanager.com/CL0/http:%2F%2Fwww.plosone.org%2Fstatic%2Fpolicies.action%23sharing/1/010f0198c191d50b-ad342c4c-4f89-4237-abf4-3b6715638fe4-000000/q0MlCJZbntA9cf21swBWr0n0Kqzwh1NpS8tdyDnwkd8=224) requires authors to make all data underlying the findings described in their manuscript fully available without restriction, with rare exception (please refer to the Data Availability Statement in the manuscript PDF file). The data should be provided as part of the manuscript or its supporting information, or deposited to a public repository. For example, in addition to summary statistics, the data points behind means, medians and variance measures should be available. If there are restrictions on publicly sharing data—e.g. participant privacy or use of data from a third party—those must be specified.

**Reviewer #1:** Yes

4. Is the manuscript presented in an intelligible fashion and written in standard English?

PLOS ONE does not copyedit accepted manuscripts, so the language in submitted articles must be clear, correct, and unambiguous. Any typographical or grammatical errors should be corrected at revision, so please note any specific errors here.

**Reviewer #1:** Yes

5. Review Comments to the Author

Please use the space provided to explain your answers to the questions above. You may also include additional comments for the author, including concerns about dual publication, research ethics, or publication ethics. (Please upload your review as an attachment if it exceeds 20,000 characters)

**Reviewer #1:** The article titled “The Apple Detection Method Based on Multimodal Features” presents a very interesting and well-structured study. The authors provide a clear and insightful contribution to the field, particularly in the application of multimodal feature integration for fruit detection, which is both timely and relevant.

The methodology is sound, and the results are presented clearly. The use of multiple data modalities enhances detection accuracy, and the analysis demonstrates the potential of the approach in practical agricultural applications. The paper offers a valuable perspective and advances understanding in this area.

While the overall quality of the work is commendable, I have suggested minor corrections to improve clarity and precision in a few sections of the manuscript. These suggestions are included in the attached file.

Response: Thank you very much for your positive recognition of our study titled “The Apple Detection Method Based on Multimodal Features” and your affirmation of its contribution, methodological soundness, and practical relevance in agricultural applications. We greatly appreciate your valuable feedback, as it encourages us to further refine our work.

We fully agree with the minor correction suggestions aimed at enhancing the manuscript’s clarity and precision, which are detailed in your attached file. We have carefully addressed each suggestion, made corresponding revisions to the relevant sections, and tracked these changes with clear markers for easy reference. Below is our specific response to the comments in the attachment:

**Comments**

**Reviewer #1:**

Line 6

change “improve the enhances” to “improve the detection” or “enhance the performance” , not both.

Response: Thank you for your careful review. We have revised the expression in Line 6 as suggested: the original "improve the enhances" has been corrected to "improve the detection" to ensure grammatical accuracy and clarity of meaning.

**Reviewer #1:**

Line 5

please add recent year report data.

Response: Thank you for your suggestion. We have supplemented recent-year data as requested. After checking official statistical sources, we note that the 2025 data has not yet been released due to the unfinished calendar year, so we adopted the latest available official data for 2024. The revised content is as follows: "According to data from the Chinese National Bureau of Statistics, China's apple production in 2024 reached 51.2851 million tons, ranking first in the world."

**Reviewer #1:**

Line 7-9

Break into two concise sentences.

Response: Thank you for your suggestion to enhance conciseness. We have split the original content in Lines 7-9 into two concise sentences as requested. The revised version is as follows: "However, the adaptability of existing image recognition technology in complex environments remains significantly lacking. This limitation restricts its practical effectiveness in agricultural scenarios."

**Reviewer #1:**

Line 10 -19

This paragraph provides useful background on traditional methods but can be made more concise.

Response: Thank you for your suggestion to enhance conciseness. We have streamlined the background content on traditional methods in Lines 10-19 while retaining key information. The revised version is as follows: "Traditional fruit recognition methods primarily rely on artificial features such as texture, color, and shape. For instance, some researchers have combined these features with relevant models to identify apple parts, but such methods depend on manually designed features and perform poorly in complex environments."

**Reviewer #1:**

Line 18

Please explain what kind of uncertainty.

Response: Thank you for your suggestion to clarify the type of uncertainty. We have supplemented specific explanations for the uncertainty mentioned in Line 18. The revised content is as follows: " Changes in lighting can distort colors and textures, leading to uncertain segmentation results, such as misclassifying shadows as fruit regions or producing incomplete outlines."

**Reviewer #1:**

Line 40

Clarify what “organ segmentation”. Does it mean fruit vs. stem?

Response: Thank you for your question regarding the definition of "organ segmentation" in Line 40. This term, cited from Reference 11, specifically refers to the segmentation of apple tree organs, including branches, buds, leaves, and their connecting parts (rather than distinguishing between fruit and stem). We have clarified this definition in the revised manuscript to avoid ambiguity.

**Reviewer #1:**

Line 50

Good transition, but could add a brief example of a depth camera.

Response: Thank you for your suggestion to enrich the content with a specific example. We have added a brief example of a depth camera to the transition sentence in Line 50 as requested. The revised version is as follows: " With the rapid development of machine vision and sensor hardware in recent years, depth cameras like the Microsoft Kinect have been widely used in fruit and vegetable recognition and positioning research."

**Reviewer #1:**

Line 72

Please merge with the previous sentence for better flow.

Response: Thank you for your suggestion to improve the text flow. We have merged the content in Line 72 with the previous sentence as requested, resulting in a more cohesive expression. The revised version is as follows: "In addition, in order to improve the level of agricultural automation and enhance the operational accuracy of robot picking systems, providing accurate spatial information and fruit location remains a significant challenge."

**Reviewer #1:**

Line 106

The apples images change to apple images it should be singular when use like this.

You may add the reason for choosing this specific camera.

Response: Thank you for your careful review and valuable suggestions. We have addressed the two points regarding Line 106 as follows:

1. We corrected the grammatical error by revising "apples images" to "apple images" to ensure accuracy.
2. We added an explanation for selecting the specific camera: Given the excellent environmental adaptability and functionality of this camera, which are well-suited for the data collection needs of this study, it was selected for the relevant data collection work.

Corresponding revisions have been made in the relevant section of the manuscript.

**Reviewer #1:**

Line 108

Nice description, but could explain why its versatile.

Response: Thank you for your suggestion to clarify the camera’s versatility. We note that the original sentence in Line 108 already mentions the camera’s ability to capture color images, depth images, and point cloud images. These multi-type image acquisition capabilities are precisely what reflect its "versatility".Specifically, its ability to meet the diverse data needs of our multi-modal study.

**Reviewer #1:**

Line 115

Please explain how these models differ visually from real apples.

Response: Thank you for your suggestion to clarify the visual difference between the apple models and real apples. We have revised the description in Line 115 to address this.

In fact, the apple models used in the study closely resemble real apples in terms of appearance and texture, with no significant visual differences. The revised content is as follows: "Some apple images were taken using apple models (these models closely resemble real apples in appearance and texture)."

**Reviewer #1:**

Line 119

Not all readers may know RGB8, please explain.

Response: Thank you for your suggestion to clarify technical terms for readers. We have added explanations for both "RGB8" and "Z16" in Line 119 to enhance accessibility, with the revised content as follows:

1. For the color format: "The images are stored in RGB8 color format (RGB8 is an 8-bit color format for each red, green, and blue channel)."
2. For the depth format: " The depth images are formatted in Z16 (Z16 is a 16-bit format for storing depth information)."

These additions ensure readers unfamiliar with these technical specifications can fully understand the image storage details, while maintaining the conciseness of the original content.

**Reviewer #1:**

Line 124

Good job explaining why 30 frames are used. This strengthens the reasoning.

Response: Thank you for your positive feedback on the 30-frame explanation. Your recognition helps confirm the rigor of this reasoning, and we will keep this clear, logical style in subsequent manuscript revisions.

**Reviewer #1:**

Line 127

You wrote 30 frames were merged but did not explain how. Please mention the software/methods.

Response: Thank you for your valuable suggestion to clarify the method of merging 30 frames in Line 127. We have supplemented detailed information about the implementation approach and technical logic in the revised manuscript, and adjusted the description of image acquisition as follows:

To address the limitation of incomplete point cloud data caused by single-frame acquisition, we adopted a near-simultaneous multi-frame accumulation method. The 30 frames used for integration were captured by the camera under program control. This program enables the camera to acquire these 30 frames almost synchronously, ensuring all frames correspond to the same 3D spatial scene of the target. During data integration, all 3D points extracted from the 30 frames are directly aggregated into a unified point cloud dataset: points overlapping in 3D spatial coordinates across frames are retained as a single point (without mutual interference), while non-overlapping points supplement spatial information coverage. This approach effectively enhances point cloud density and completeness without the need for complex coordinate alignment or fusion algorithms, as the near-simultaneous acquisition ensures spatial consistency across frames.

**Reviewer #1:**

Line 139

Voxel Down Sampling technology consider simplifying.

Response: Thank you for your suggestion to simplify the description of Voxel Down Sampling in Line 139. We have revised the content to make it more concise while retaining key technical details. The updated version is as follows: "This research employs Voxel Down Sampling technology to reduce point cloud noise. This technique discretizes the point cloud into a voxel grid (each voxel representing a small spatial cube)."

**Reviewer #1:**

Line 157

Zeros are applying Explain why it is important.

Response: Thank you for your suggestion to clarify why zeros are applied in Line 157. We have revised the content to clearly explain its importance while keeping the core meaning intact. The updated version is as follows: "After noise removal, zeros are applied to fill empty regions in the point cloud to ensure data integrity."

**Reviewer #1:**

Line 161-163

Add one sentence about why this is important.

Response: Thank you for your guidance to add a sentence explaining the importance of this step in Lines 161-163. We have supplemented the relevant content as follows: "Specifically, such accurate spatial features ensure precise alignment between 3D and 2D visual data in multimodal fusion, preventing target missed or false detection and supporting better target detection accuracy." We have made the corresponding revisions in the manuscript.

**Reviewer #1:**

Line 183

Consider explaining why 80% was chosen as the cutoff. Is this standard?

Response: Thank you for your valuable suggestion. The 80% occlusion threshold was determined based on two key considerations. First, it is a practical choice to balance data retention and label reliability. This cutoff helps avoid keeping overly ambiguous samples—those lacking clear fruit feature cues—since such samples would provide confusing information and mislead the model during training. Second, our tests found that when fruit occlusion exceeds 80%, the remaining visible features (e.g., shape, color texture) are too limited. They cannot give the model effective information to learn discriminative characteristics. If we included these highly occluded samples, the model would struggle to capture consistent, recognizable fruit features, which would directly reduce training efficacy. In short, this 80% threshold strikes a practical balance between preserving usable data and ensuring annotation quality.

**Reviewer #1:**

Line 189

fruits occluded heavily reword it for smooth flow.

Response: Thank you for your valuable suggestion. We note that the relevant content about "fruits occluded heavily" you mentioned is in Line 182. We have revised it for smoother flow as follows: "During the annotation process, fruits with heavy occlusion are omitted, specifically when the obscured area of a fruit exceeds 80% of its total area."

**Reviewer #1:**

Line 282

The feature map of color difference… this line is too long.

Response: Thank you for your valuable suggestion. We have split the long sentence in Line 282 for better readability. The revised content is as follows: "The feature map of color difference is an intuitive graphical representation of color features. By highlighting luminance disparities among regions in an image, it effectively separates fruits from the background."

**Reviewer #1:**

Line 287

Suppressed could be simplified to reduced.

Response: Thank you for your valuable suggestion regarding Line 287. We have revised the word "Suppressed" to "Reduced" as recommended, aiming for a more concise expression. Corresponding adjustments have been made in the manuscript.

**Reviewer #1:**

Line 289

Use simple language.

Response: Thank you for your valuable suggestion to use simpler language in Line 289. We have revised the content accordingly, and the updated version is as follows:"However, some parts of fruits may be missed in complex lighting."

**Reviewer #1:**

Line 325

Correct grammar.

Response: Thank you for pointing out the grammar issue in Line 325. We have revised the sentence as follows:"These color difference maps and edge maps are merged based on a predefined weight ratio to generate a key fruit feature map."

Corresponding corrections have been made in the manuscript.

**Reviewer #1:**

Line 329

You could specify how many channels are in final image.

Response: Thank you for your valuable suggestion. We have revised the sentence as follows:"Finally, the R, G, B channels, key fruit feature map, depth information and point cloud coordinate data (XYZ) are combined to create an image dataset with 8 channels."

**Reviewer #1:**

Line 390 to 395

Very detailed settings but you can convert it into a table or appendix.

Response: Thank you for your valuable suggestion. The revised content is as follows:

To evaluate the performance improvements in fruit detection using the refined YOLOv5 model framework with different feature fusion strategies, a comprehensive series of experiments was conducted for comparative analysis. The key experimental settings are detailed in Table 1 below, ensuring clarity and accessibility for reference.

**Table 1． Key Experimental Settings.**

| **Category** | **Specific Configuration** |
| --- | --- |
| Software Platform | PyCharm Community Edition |
| Hardware Setup | Intel(R) Core(TM) i5-11400H CPU; 16GB RAM; 1× RTX 3050 GPU (12GB dedicated memory) |
| Optimization Algorithm | Stochastic Gradient Descent (SGD) |
| Training Batch Size | 4 (constrained by hardware limitations) |
| Initial Learning Rate | 0.01 |
| Learning Rate Decay Factor | 0.01 |
| Momentum Coefficient | 0.937 (0.8 during warm-up phase) |
| L2 Regularization Factor | 0.0005 |
| Warm-up Phase | 3 epochs (bias learning rate fixed at 0.1 during this period) |
| Maximum Training Epochs | 200 |

**Reviewer #1:**

Line 420

table number 1

Mention units clearly

Response: Thank you for your valuable suggestion. We have revised the table accordingly.

**Reviewer #1:**

Line 474 to 476

You can emphasize it why this combination is powerful.

Response: Thank you for your valuable suggestion.  We have supplemented the relevant section, and the added content is as follows:

"By integrating RGB images, key fruit features, depth information, and point cloud coordinates at the input layer, the model demonstrates superior performance in complex environments, as this combination leverages the complementary strengths of each modality: RGB and key features provide discriminative visual cues, while depth and point cloud data supply spatial-structural context to compensate for single-modal limitations."

**Reviewer #1:**

Line 494 495

Also explain How this helps in agriculture.

Response: Thank you for your valuable suggestion. We have revised this section, and the revised content is as follows:

"The model demonstrated superior performance in complex lighting conditions with varying intensities, achieving 95.8% accuracy, 96% recall, and an F1 score of 95.9% in apple recognition tasks. Specifically, it enhances the efficiency of robotic harvesting and lowers labor costs in agricultural applications."

**Reviewer #1:**

1.What is the purpose of Author summary section after abstract? Why it is included?

Response: Thank you for your valuable suggestion. We have removed this section.

**Reviewer #1:**

2.Remove the guidelines added in start of materials and methodology section. check every section carefully.

Response: Thank you for your valuable suggestion. We have removed this section and conducted a check on each chapter.

**Reviewer #1:**

3. The information in few sections is added like a lab procedure. for instance Data processing. Improve wriitng.

Response: Thank you for your valuable suggestion. We have made revisions to the relevant sections.

**Reviewer #1:**

4. Remove basic and/or well known information. Rewrite carefully.

Response: Thank you for your valuable suggestion. We have made revisions to the relevant sections.

**Reviewer #1:**

5. The 182 lines "During the annotation process, the fruits occluded heavily are omitted, specifically when the obscured area of the fruit surpasses 80% of its total area..." How will it handle the real conditions? How occlusion problems can be addressed?

Response: Thank you for your valuable suggestion. During the annotation process, fruits with heavy occlusion are omitted, specifically when the obscured area of a single fruit exceeds 80% of its total area. This annotation protocol is designed to ensure the reliability of training data by excluding ambiguous samples that may introduce noise into model learning, while also establishing alignment with real-world application scenarios. In practical automated fruit-picking tasks, highly occluded fruits (e.g., those fully obscured by foliage or overlapping conspecifics) are typically inaccessible to picking mechanisms, rendering their detection of limited practical utility for operational workflows.

To further address occlusion challenges in real-field conditions, the proposed multimodal fusion framework serves as a core technical solution. By fusing RGB imagery (capturing surface texture and chromatic features), depth information (delineating inter-object spatial hierarchies), and point cloud coordinates (reconstructing three-dimensional structural attributes), the model enables inference of the complete contour and spatial position of partially occluded fruits (with occlusion degree below 80%) via complementary modal cues. For example, when a fruit’s edge features are occluded in RGB data, the depth map can still discriminate its spatial separation from surrounding vegetation, while the point cloud supplements missing structural details. This synergistic integration of multimodal data mitigates missed detection and false detection arising from partial occlusion, thereby enhancing the framework’s adaptability to complex, cluttered real-orchard environments.

**Reviewer #1:**

6. Add section and sub-section numbering. Improve writing and flow.

Response: Thank you for your valuable suggestion. We have made the corresponding revisions.

**Reviewer #1:**

7. How the anchor boxes are selected? Based on which previous experience as said?

Response: Thank you for your valuable suggestion. The selection of anchor boxes follows a two-stage process: initial adoption and targeted optimization.

First, nine initial anchor box dimensions (10×13, 16×30, 33×23, 30×61, 62×45, 59×119, 116×90, 156×198, 373×326) are adopted based on the default anchor configurations of the original YOLOv5 framework—a prior experience widely validated in general object detection tasks, especially for objects with size distributions similar to the fruit targets in this study.

Next, the initial anchors’ suitability for the specific fruit dataset is evaluated using two metrics: *Anchors Above Threshold* (proportion of anchors overlapping annotated boxes beyond a pre-defined threshold) and *Best Possible Recall* (maximum recall of current anchors). If compatible, no recalculation is needed; otherwise, k-means clustering (with 30 iterations and data normalization) is used to cluster the width/height of all annotated boxes in the training dataset, generating dataset-adapted anchors. These anchors are further optimized via a genetic algorithm (iterative adjustment through random mutation and fitness evaluation, with fitness based on anchor-true box width-height ratio) to enhance small-object detection, and excessively small anchors are filtered out via a threshold.

**Reviewer #1:**

8. Upload high quality images inside text file for proper evaluation. The attached images at the end are not clear.

Response: Thank you for your valuable suggestion. We have made changes to the relevant sections. To prevent the images in the manuscript from being compressed, we have also attached the corresponding images at the end of this document.

**Reviewer #1:**

9. How the model robustness against lighting variations and background noise is increased?

Response: Thank you for your insightful question. To enhance the model’s robustness against lighting variations and background noise, we adopted two targeted strategies based on our study design:

1. **Training with diverse lighting intensities**: Our dataset includes apple images captured under a wide range of real-world lighting conditions, such as strong sunlight, overcast weather, and low-light shade. This allows the model to learn features of apples that remain consistent regardless of lighting (like their inherent shape and texture) during training. Instead of relying on unstable cues that change with light (such as temporary brightness or shadows), the model becomes less prone to performance drops when lighting conditions vary.
2. **Multi-modal fusion with light-insensitive features**: The modified YOLOv5 framework integrates not only RGB images (which are sensitive to lighting) but also multi-modal features that are less affected by illumination—specifically depth features (D) and point cloud coordinate features (XYZ). Depth information reflects the spatial distance of apples, while point cloud coordinates capture their 3D structural attributes; both are inherently robust to lighting fluctuations and background noise. These features complement RGB data, helping the model effectively distinguish apple targets from cluttered, light-disturbed backgrounds and maintain stable detection performance.

Together, these two strategies work in synergy to strengthen the model’s ability to adapt to varying lighting environments and resist background noise, ensuring reliable apple recognition in natural orchard settings.

**Reviewer #1:**

10. Compare inference results with other models.

Response: Thank you for your valuable suggestion on comparing inference results with other models. We have supplemented a comparative analysis of our improved YOLOv5 with other representative object detection models in the revised manuscript, and the detailed performance data is presented in Table 6.

All models were evaluated under the same experimental conditions: they used the identical multi-modal dataset and adopted the 8-channel input configuration of RGB+XYZ+D+F, ensuring the fairness of the comparison. Specifically, we selected two mainstream models as benchmarks: the single-stage SSD + VGG16 and the two-stage Faster RCNN + VGG16. From Table 6, it can be observed that the SSD + VGG16 achieves a precision of 86.6%, recall of 78%, and F₁ score of 82.08%; the Faster RCNN + VGG16 shows improved performance, with precision reaching 88.2%, recall 81%, and F₁ score 84.45%.

In contrast, our improved YOLOv5 outperforms both models significantly, achieving a precision of 95.8%, recall of 96%, and F₁ score of 95.9%. This result confirms that under the multi-modal input and dataset conditions of this study, our improved YOLOv5 has distinct advantages in object detection. It can more accurately recognize and localize targets, thus delivering better overall detection performance.

We hope this supplementary comparative analysis addresses your concern adequately.

**Reviewer #1:**

11. Test the model on unknown/new data.

Response: Thank you for your valuable suggestion on testing the model with unknown/new data. We addressed this aspect through two key considerations aligned with our multi-modal dataset design and availability.

1. **regarding the evaluation on unseen data:** Before conducting experiments, our multi-modal dataset (including RGB, depth, and point cloud data) was pre-divided into three independent subsets: training set, validation set, and test set. Notably, the test set consists entirely of samples collected through multiple separate capture sessions (e.g., different days, varying orchard locations, and adjusted camera angles) that were never involved in model training or validation. These test samples thus represent "new/unknown data" the model had not encountered during its learning process, ensuring we could assess generalization to unseen scenarios.
2. **regarding the scarcity of matching public data**: As our model relies on multi-modal input (rather than single RGB images), publicly available datasets that include paired RGB, depth, and point cloud data for apple detection are extremely limited. This makes it challenging to use external unknown datasets for additional testing. However, our pre-split test set with its independently collected, unseen multi-modal samples serves as a practical and rigorous alternative to validate the model’s ability to handle new data. The stable performance observed on this test set confirms the model’s generalization capability in real-world multi-modal scenarios.

**Reviewer #1:**

12. The model section shares irrelevant information. How YOLOv5 is improved? What are the architectural/layer changes as mentioned?

Response: Thank you for your valuable suggestion. We have refined the model section by removing irrelevant information and supplemented clear descriptions of YOLOv5’s improvements and corresponding architectural/layer changes, as detailed below:

To address the need for integrating multi-modal fruit data, we made targeted architectural modifications to the original YOLOv5 framework, with all improvements focused on the input layer (as depicted in Fig. 8). The specific changes are twofold:

1. **Input channel expansion**: The original YOLOv5 input layer only supports 3-channel RGB images, so we adjusted it to an 8-channel configuration to accommodate multi-source feature data. These 8 channels include conventional RGB images, key fruit features (F), depth feature information (D), and point cloud coordinates information (XYZ)，matching the 8-channel image structure of dataset DA2 used in this research.
2. **Input-layer convolutional kernel adjustment**: The convolutional kernel of the input layer follows the dimension form W×H×m×n (W×H denotes the kernel’s length and width, m represents the number of kernel layers matching input channels, n represents the number number of feature extraction kernels). In the original network, W×H is 6×6 and n is 32; to align with the 8-channel input, we only recalibrated m from 3 (for 3-channel RGB) to 8, while keeping W×H and n unchanged to ensure stable basic feature extraction.

We believe this revised content clearly answers how YOLOv5 was improved and specifies the architectural/layer changes, while eliminating redundant information. We hope this addresses your concern adequately.

**Reviewer #1:**

13. The underperformance due to information redundancy is not analyzed?

Response: Thank you for your valuable observation. We have supplemented the analysis of information redundancy leading to underperformance in the revised manuscript. Specifically, for the RGB+(R-G)+E+D configuration, we explain that chromaticity features (R-G) and edge features (E) overlap with the inherent color and structural information of RGB images, while depth features (D) introduce spatial data that conflicts with these visual cues, creating redundant and interfering signals that confuse the model's feature learning. This detailed analysis clarifies how information redundancy and interference contribute to the configuration's underperformance, emphasizing the critical role of rational feature selection and fusion strategies in model design. We hope this revision addresses your concern adequately.

**Reviewer #1:**

14. The yolo variants are compared but the training/comparison conditions are not provided? Is it architectural improvement?

Response:Thank you for your valuable suggestion. Regarding the comparison of YOLO variants, we ensured consistent training and evaluation conditions to fairly assess their performance on apple recognition, while the performance differences observed primarily stem from architectural improvements of each variant. Here are the detailed explanations:

1. **Unified training and comparison conditions**

All models (YOLOv3, YOLOv5, YOLOv6, YOLOv8) were trained and tested under identical experimental settings to eliminate interference from non-architectural factors:

- **Dataset consistency**: All models used the *same color image subset from dataset DA2* (with unified image resolution, annotation format, and train-test split ratio of 8:2) to avoid dataset bias affecting performance comparisons.
- **Model version alignment**: Given the task’s demand for real-time inference (requiring a balance between speed and accuracy), we selected the *small-sized ("s") variant* of each YOLO model—this ensures consistency in model scale and parameter count magnitude, avoiding unfair advantages from larger model sizes.
- **Hyperparameter and training protocol unification**: All models adopted the same training configuration: stochastic gradient descent (SGD) as the optimizer, initial learning rate of 0.01, batch size of 4 (constrained by hardware consistency), 200 training epochs, and identical data augmentation strategies (random horizontal flip, brightness adjustment, and crop). No additional optimization (e.g., custom loss functions or pre-trained weights fine-tuning beyond the official ImageNet initialization) was applied to any model, ensuring that performance variations were not caused by training procedure differences.

2. **Performance differences originate from architectural improvements**

The superior performance of YOLOv5 (among "s" variants) on apple recognition is attributed to its architectural optimizations compared to earlier/later YOLO variants:

- Compared to YOLOv3: YOLOv5 uses a more efficient backbone (CSPDarknet instead of Darknet-53) with cross-stage partial connections to reduce redundant computations while retaining feature integrity; its neck module (SPP and PANet) enhances multi-scale feature fusion, which is critical for capturing apple targets of varying sizes in orchard scenes.
- Compared to YOLOv6: YOLOv5’s "s" variant adopts a more lightweight head design. While YOLOv6 focuses on anchor-free detection, its "s" version has higher computational complexity in feature mapping, leading to slightly lower inference efficiency and higher parameter count than YOLOv5-s—this aligns with our observation that YOLOv5-s has the smallest parameter count under the same task requirements.
- Compared to YOLOv8: YOLOv8’s "s" variant introduces more complex feature pyramid structures, but its increased parameter redundancy (relative to YOLOv5-s) does not translate to better apple recognition accuracy in our task; this may be due to YOLOv5’s architecture being more compatible with the texture and shape characteristics of apple targets, as its feature extraction module is more focused on local detail preservation (e.g., apple surface texture and edge contours).

In summary, the consistent training conditions ensure the fairness of the YOLO variant comparison, and the observed performance advantages of YOLOv5 confirm that its architectural design is more suitable for the real-time apple detection task in this study.

**Fig 1. Apple Color Image and Pseudo-Color Depth View Set.** (a) Color Image of Apple, and (b) Apple Pseudo-Color Depth Image.

**Fig 2. Apple Point Cloud Generated by Python Script.**

**Fig 3. Apple Images Captured under Different Light Intensity Conditions.** (a) Apple Image under Light Intensity a, (b) Apple Image under Light Intensity b, (c) Apple Image under Light Intensity c, (d) Apple Image under Light Intensity d, (e) Apple Image under Light Intensity e, (f) Apple Image under Light Intensity f.

**Fig 4. Flowchart of the Local Outlier Detection Method.**

**Fig 5. Manually Annotated and 2D-to-3D Mapped Views of Apples.** (a) Manually Annotated Apple Image, (b)2D-to-3D Mapped Apple Point Cloud Front View, (c) 2D-to-3D Mapped Apple Point Cloud Right View, (d) 2D-to-3D Mapped Apple Point Cloud Top View.

**Fig 6. Apples Under Varying Illumination Intensities: Chromaticity, Edge, and Key Fruit Feature Maps.** (a) Original apple image under lighting condition a, (b) Original apple image under lighting condition b, (c) Original apple image under lighting condition c, (d) Chroma image of apple under lighting condition a, (e) Chroma image of apple under lighting condition b, (f) Chroma image of apple under lighting condition c, (g) Edge image of apple under lighting condition a, (h) Edge image of apple under lighting condition b, (i) Edge image of apple under lighting condition c, (j) key fruit characteristic mapping of apples under light a, (k) key fruit characteristic mapping of apples under light b, (l) key fruit characteristic mapping of apples under light c, (m) Original apple image under lighting condition d, (n) Original apple image under lighting condition e, (o) Original apple image under lighting condition f, (p) Chroma image of apple under lighting condition d, (q) Chroma image of apple under lighting condition e, (r) Chroma image of apple under lighting condition f, (s) Edge image of apple under lighting condition d, (t) Edge image of apple under lighting condition e, (u) Edge image of apple under lighting condition f, (v) key fruit characteristic mapping of apples under light d, (w) key fruit characteristic mapping of apples under light e, (x) key fruit characteristic mapping of apples under light f.

**Fig 7. The flow chart of synthesizing the multi-channel images.**

**Fig 8. Architecture of the YOLOv5 Object Detection Model with Optimized Input Layer.**

**Fig 9. Pseudocode for Automatic Anchor Optimization in the YOLOv5 Model.**

**Fig 10. Recognition Performance of YOLOv5 under RGB+XYZ+D+F Configuration.** (a) Apple prediction plot under lighting condition a, (b) Apple prediction plot under lighting condition b, (c) Apple prediction plot under lighting condition c, (d) Apple prediction plot under lighting condition d, (e) Apple prediction plot under lighting condition e, (f) Apple prediction plot under lighting condition f.
